# Evaluation of a structured goal planning and tailored follow-up programme in rehabilitation for patients with rheumatic diseases: protocol for a pragmatic, stepped-wedge cluster randomized trial

**DOI:** 10.1186/1471-2474-15-153

**Published:** 2014-05-14

**Authors:** Ingvild Kjeken, Gunnhild Berdal, Ingvild Bø, Turid Dager, Anne Dingsør, Jon Hagfors, Bente Hamnes, Siv G Eppeland, Elin Fjerstad, Petter Mowinckel, Merete Nielsen, Randi W Rørstad, Anne-Lene Sand-Svartrud, Bente Slungaard, Sigrid H Wigers, Kåre Birger Hagen

**Affiliations:** 1 National Advisory Unit on Rehabilitation in Rheumatology, Department of Rheumatology, Diakonhjemmet Hospital, PO Box 23 Vinderen, N-0319 Oslo, Norway; 2 Department of Rehabilitation, Lillehammer Hospital for Rheumatic Diseases, Margrethe Grundtvigs v 6, N-2609 Lillehammer, Norway; 3 National Unit for Rehabilitation in Rheumatology, Department of Rheumatology, Diakonhjemmet Hospital, PO Box 23 Vinderen, N-0319 Oslo, Norway; 4 Department of Rheumatology, Betanien Hospital, Bj. Bjørnsonsgt 6, N-3722 Skien, Norway; 5 Norwegian Rheumatism Association, PO Box 2653 Solli, N-0203 Oslo, Norway; 6 Department of Self-management, Lillehammer Hospital for Rheumatic Diseases, Margrethe Grundtvigs v 6, N-2609 Lillehammer, Norway; 7 Department of Rheumatology, Sørlandet Hospital, Sykehusveien 1, N-4838 Arendal, Norway; 8 Department of Rheumatology, Martina Hansen’s Hospital, Bærum Postterminal, PO Box 23, N-1306 Bærum, Norway; 9 Jeløya Kurbad Rehabilitation Centre, Bråtengaten 94, N-1515 Moss, Norway

**Keywords:** Rehabilitation, Rheumatic diseases, Goal attainment, Motivational interviewing, Follow-up intervention, Stepped-wedge trial

## Abstract

**Background:**

Comprehensive rehabilitation, involving health professionals from various disciplines, is widely used as an adjunct to pharmacological and surgical treatment in people with rheumatic diseases. However, the evidence for the clinical- and cost-effectiveness of such interventions is limited, and the majority of those who receive rehabilitation are back to their initial health status six to 12 months after discharge.

**Methods/design:**

To evaluate the goal attainment, health effects and cost-effectiveness of a new rehabilitation programme compared to current traditional rehabilitation programmes for people with rheumatic diseases, a stepped-wedge cluster randomized trial will be performed. Patients admitted for rehabilitation at six centres in the south-eastern part of Norway will be invited to participate. In the trial, six participating centres will switch from a control (current rehabilitation programme) to an intervention phase (the new rehabilitation programme) in a randomized order. Supported by recent research, the new programme will be a supplement to the existing programme at each centre, and will comprise four elements designed to enhance and support lifestyle changes introduced in the rehabilitation period: structured goal-planning, motivational interviewing, a self-help booklet and four follow-up telephone calls during the first five months following discharge. The primary outcome will be health-related quality of life and goal attainment, as measured by the Patient Generated Index directly before and after the rehabilitation stay, as well as after six and 12 months. Secondary outcomes will include self-reported pain, fatigue, a global assessment of disease activity and motivation for change (measured on 11-point numeric ratings scales), health-related quality of life as measured by the Short Form 36 Health Survey (SF-36) and utility assessed by the SF6D utility index.

The main analysis will be on an intention to treat basis and will assess the clinical- and cost-effectiveness of the structured goal planning and tailored follow-up rehabilitation programme for patients with rheumatic diseases.

**Discussion:**

The findings will constitute an important contribution to more cost-effective- and evidence-based rehabilitation services for people with rheumatic diseases.

**Trial registration:**

ISRCTN91433175.

## Background

Inflammatory rheumatic diseases are characterized by inflammatory processes that may lead to joint damage and various degrees of disability, thus representing a major burden to the individual patient and society. Current treatment focuses on early diagnosis and early use of disease modifying agents. Nevertheless, a major percentage of patients has a residual disability and will need some form of rehabilitation throughout their life [1].

Rehabilitation has been defined by the World Health Organization as “a process aimed at enabling people with disabilities to reach and maintain their optimal physical, sensory, intellectual, psychological and social functional levels. Rehabilitation provides disabled people with the tools they need to attain independence and self-determination” [2].

As such, the rehabilitation process involves several professions and services, and is characterized by being tailored to the patients’ individual needs and challenges, with the aim of reducing the consequences of illness while improving activity, participation and quality of life [3].

Several studies have shown that patients with rheumatic diseases benefit from rehabilitation. However, the effect seems to fade over time, and most patients are back to their initial health status six to 12 months after discharge [4-7]. A major challenge is therefore to help patients maintain self-management strategies introduced in the rehabilitation period, thereby enhancing a longer lasting effect of rehabilitation.

Goal planning is considered as an essential part of rehabilitation practice and is a process directed at setting goals at various levels of function, within different life areas and in various time frames [8]. The key components of goal setting are comprised of goal identification and negotiation, action planning and appraisal and feedback of performance [9,10], and individualized weekly action plans and participant handbooks are recommended to enhance participants’ adherence and progress towards long-term goals [11]. Even though research concerning the effectiveness of goal planning in clinical rehabilitation is scarce, the conclusions in a systematic review by Levack et al. are that there is some, though limited, evidence that goal planning can influence patient adherence to treatment regimes, as well as strong evidence that specific, challenging goals can improve immediate patient performance in some specific clinical contexts [3]. In addition to having a potential for enhancing the individual effect of rehabilitation, knowledge about the content of rehabilitation goals in larger patient groups is important to help ensure the delivery of relevant rehabilitation interventions. Although there are some studies that have explored rehabilitation goals in other patient groups [12-15], little is known about rehabilitation goals in people with rheumatic diseases.

The objective of goal setting involves a change in patient behaviour. Hence, theories of behaviour and behavioural change may guide goal-setting interventions [16-18], and several reviews conclude that the use of cognitive behavioural approaches in exercise programmes and other self-management interventions increase their effectiveness in people with rheumatic diseases [11,19-21]. Reviews have further demonstrated that motivational interviewing (MI) is an effective approach towards changing behaviour [22-26]. MI is a client-centred information and motivation strategy based on cognitive behavioural theory and the trans-theoretical model [27,28]. It is designed to engage ambivalent or resistant clients in the process of health behaviour change, and also provides health practitioners with a means of tailoring their interventions to suit the patient’s degree of readiness for change. The approach has been widely used in various clinical conditions such as substance abuse, dietary adherence and smoking cessation, and may be an effective strategy to also promote healthy lifestyle changes in people with rheumatic diseases.

Rehabilitation often addresses health lifestyle changes involving a process that takes a certain amount of time before being settled in as a new habit. The process involves both cognitive and behavioural elements that may by the end of a rehabilitation stay still be unclear and fragile [29]. A follow-up intervention may therefore provide psychological and social support to individual patients, thus helping them achieve individual rehabilitation goals, implement lifestyle changes and adhere to different treatment recommendations. Three randomized controlled trials (RCTs) have demonstrated that patients who received telephone calls to support individualized goals and action plans had an increased adherence to- and effect of the programme [30-32]. However, to the best of our knowledge, active support after patients have been discharged and returned to their local communities is not yet a part of routine rehabilitation care in Norway, which may to some extent explain why the effect of rehabilitation fades within a few months.

The treatment and rehabilitation of complex chronic conditions are often resource-intensive and expensive, both for the individual patient and for society [33], with musculoskeletal disease accounting for the largest proportion of health- and social benefits payments in Norway [34]. From a health economic perspective, a follow-up intervention may be cost saving, as it may prevent exacerbations of the disease, reduce non-adherence to treatment and intensify or prolong the initial treatment investment by helping people cope with chronic illness and manage disease consequences. This may reduce the burden of illness, restrict revisits to clinicians and reduce treatment costs, prevent readmissions to hospitals or rehabilitation centres and reduce the general health-care expenditures [35,36]. If effective, follow-up interventions may both save costs and increase and provide a longer lasting effect of treatment and rehabilitation [30-32].

### The aims and research questions of this study

The main objective will be to evaluate goal attainment, health effects and the cost-effectiveness of a new rehabilitation programme (which includes structured goal planning and tailored follow-up after discharge) compared to current traditional rehabilitation programmes (with no structured goal planning or tailored follow-up) for patients with rheumatic diseases.

More specifically, our study will consider the following research questions:

• What is the content of patients’ rehabilitation goals throughout the rehabilitation and follow-up period, and are the goals stable over time?

• Is the new rehabilitation programme more effective with regard to goal attainment and an improved health-related quality of life compared to traditional rehabilitation programmes for patients with rheumatic diseases?

• Which factors (personal, disease-related and contextual) are associated with goal attainment and an improved health-related quality of life during the first year following rehabilitation?

• Which of the two programmes (rehabilitation with structured goal planning and tailored follow-up after discharge vs. traditional rehabilitation with no structured goal planning and tailored follow-up) provides the most cost-effective use of health-care resources?

## Methods

### Study development

The trial will be developed as part of the research activity in a Norwegian regional rheumatology research network in Health Region South-East during the period from 2010–2012, and is funded by a grant from the Regional Health Authority. The rehabilitation branch in the network consists of three clinicians from the National Advisory Unit on Rehabilitation in Rheumatology, six local study coordinators from five departments of rheumatology and one rehabilitation centre, respectively (hereafter termed centres), in addition to three representatives from the Norwegian Rheumatism Association. The group is led by two researchers from the National Advisory Unit on Rehabilitation in Rheumatology (KBH and IK). From March 2011, a research coordinator also works full-time in the project (GB). The group will meet five times during 2010 and the spring of 2011 to develop the trial, including the designing and piloting of the new rehabilitation programme. Additionally, e-mail correspondence, telephone meetings and separate meetings with two of the patient representatives will be used to exchange information and give feedback on protocol and intervention material.

### Study design

**Figure 1:**
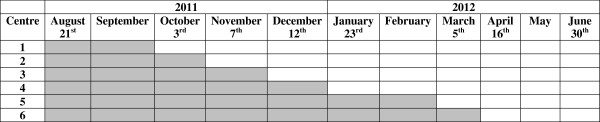
**The stepped wedge randomised controlled design.** The inclusion of participants will start August 21^st^ 2011, and end June 30^th^ 2012. White cells represent intervention periods and grey cells represent control periods. Dates for switching from control period to intervention period are recorded in head of the column.

This will be a pragmatic, multicentre, stepped-wedge cluster randomized controlled trial [37,38] in which participants will be assessed at admission to- (baseline) and discharge from a rehabilitation stay, and after six and 12 months (Trial registration: ISRCTN91433175).In the trial, six participating centres (clusters) will switch from the control- (the current rehabilitation programme) to the intervention phase (a new rehabilitation programme with structured goal planning and tailored follow-up added to the existing programme) in a randomized order (see Figure 1 for an outline of the study design). All centres will start the trial simultaneously and act as controls until the point in time that they are randomized to cross over from control to intervention, and all centres will provide the new programme by the end of the inclusion period.

The new programme will represent a supplement to the existing rehabilitation programmes, and all patients will, regardless of being included in the trial or not, receive the programme currently delivered at the different participant institutions when they are admitted. However, patients admitted in the intervention phase who do not participate in the trial will not receive a telephone follow-up after being discharged.

### Participants and selection criteria

A minimum of 312 participants will be included in the period from August 21^st^ 2011 to June 30^th^ 2012 through an invitation from health professionals at each participating centre on admission to the rehabilitation stay. Those who are interested will be screened for eligibility, and receive oral and written information about the study, and to enrol, participants will have to give their written informed consent.

People will be eligible if they are 18 years or older, are admitted for rehabilitation at one of the six participating centres, have a good understanding of the Norwegian language and have one of the following inflammatory rheumatic diagnoses: rheumatoid arthritis, psoriatic arthritis, ankylosing spondylitis, systemic lupus erythematosus and juvenile idiopathic arthritis. In addition, patients with generalized osteoarthritis with an affection of the hip and/or knee will be included. Each participant’s diagnosis will be verified by a clinical examination by a physician at admission, according to the diagnostic criteria for each of the specific diagnoses. The exclusion criteria will be cognitive impairment, severe psychiatric disorder or being admitted for rehabilitation after elective orthopaedic surgery.

### Sample size calculations

The sample size and power calculations have been estimated for the primary end point in this study by use of the sum score of the Patient Generated Index (PGI) at the six- and 12-month follow-ups. Based on results from a previous study [39], it is estimated that a sample size of six clusters at 80% power, with an average of 52 individuals per cluster (total of 312), will be needed to detect a mean 10% difference (=10 points) between the groups when the standard deviation is approximately 14 units in the baseline PGI scores, the intra-cluster correlation is 0.3 and the drop-out rate is 20%.

### Randomization and blinding

According to the stepped-wedge design, the intervention will be sequentially rolled out at the participating centres over a number of different time periods (see Figure 1 for an outline of the study design). The order in which the different centres will receive the intervention will be determined at random by the study biostatistician (PM) by using a computer-generated random numbers programme (SAS). The randomisation procedure will take place in august 2010, and will be made known to the participating centres at a workshop arranged immediately before starting inclusion of participants. The randomisation group of each participant will be determined by her/his admission date; i.e. consenting patients admitted to a centre delivering the control intervention will be included in the control group, whereas patients admitted to a centre delivering the new intervention will be included in the intervention group.

As with most non-pharmacological treatments, it will be impossible to carry out a double blind study because the therapists will know which programme they will deliver.

The new programme will represent a supplement to- and an extension of the existing rehabilitation programmes, and all patients will, regardless of being included in the trial or not, receive the programme currently offered at the different participant institutions. As opposed to other randomized controlled trials in which participants are randomized into either a treatment- or control group, we therefore believe that this design may keep participants blinded for group allocation.

The outcome data at the six- and 12-month follow up will be collected in telephone interviews by a secretary who is blinded for the allocation sequence.

Furthermore, the statistician who will perform the main statistical analyses will be blinded to group allocation during analyses.

### Training of intervention providers

Four workshops will be arranged in the data collection period of the study to prepare and educate the providers of the intervention at each centre, and the first workshop will be held before the start of the study with an agenda comprising the following lectures and exercises:

• The theoretical basis of motivational interviewing (MI);

• Exercises in how to practice MI in setting rehabilitation goals;

• Using MI in telephone follow-up calls;

• Content and use of the self-management booklet;

• Logistics, routines for data collection and management of data in the study;

• Exercises in how to invite and inform study participants.

The second workshop will be held at each participating centre a few weeks before the centre switches from the control to the intervention period. Three persons from the National Advisory Unit on Rehabilitation in Rheumatology (TD, GB and IK) will visit the centre and give lectures and training sessions in project organization, the methods of data collection, privacy matters and MI.

Half-way through the data collection period, a third workshop with all participating centres and intervention providers, comprising more lectures and exercises in MI, setting rehabilitation goals, telephone follow-up calls and study logistics will be held. Additionally, providers from the centres that have switched to the new rehabilitation programme will share their experiences, and there will also be time for questions and group discussions related to relevant topics.

The last workshop will be arranged when all telephone follow-up calls have been completed. The main aim of this workshop will be to allow for feedback from intervention providers to researchers and vice versa, and to discuss ideas and methods for implementing the new programme into clinical practice.

All local study coordinators, intervention providers and representatives from the Norwegian Rheumatism Association (NRA) will be invited to participate in the first, third and fourth workshop, and the local study coordinator and a local representative from the NRA will be invited to participate at the workshops held at each centre.

### Interventions

Both groups will receive medical treatment as usual.

#### Control group

The control group will receive the traditional rehabilitation programme provided at each participating centre at the start of the study (see Table 1 for a brief description of the programme at each centre). Directly following the second (local) workshop, we will interview the local study coordinator, using the STAR-ETIC rehabilitation framework to structure the interview and capture the context, structure, process and outcomes of the current rehabilitation programme [40].

**Table 1:** Description of the traditional rehabilitation programme (control intervention) provided at each participating centre at the start of the study

**Centre**	**Structure of the rehabilitation stay**			**Interventions (length of sessions and number of sessions pr week)**		
	**Duration and setting**	**Professions in the rehabilitation team**	**Team meetings**	**Group sessions**	**Individual sessions**	**Un-supervised sessions**
1	Three to four weeks in-patient rehabilitation (5 days with active treatment pr week)	Rheumatologist, physician, PT, OT, nurse	Twice pr week	Pool exercises (30 min × 5)	PT (30 min × 4)	Nordic walking, biking and/or pool exercising (as desired)
					OT (as needed)	
				Gym exercises (30 min × 5)	Psychologist (as needed)	
				Gym exercises (60 min × 4)		
				Patient education (60 min × 1)		
2	Two weeks at a day hospital (5 + 5 days with active treatment)	Physician, PT, OT, SW	Once pr week	Pool exercises (30 min × 5)	PT, OT or SW (30 min × 5)	Pool (30 min x 5)
				Hand exercises (30 min × 5)		
				Gym exercises (45 + 45 min × 5)		
3	Two weeks in-patient rehabilitation (5 + 5 days with active treatment)	Rheumatologist, physician, PT, OT, nurse	At admission and discharge	Pool exercises (30 min × 5)	PT (45 min × 4)	Gym exercising (as desired)
					OT (as needed)	
				Gym exercises with moderate intensity (30 min x 5) and/or	Nurses (as needed)	
				Gym exercises with high intensity (30 min x 5)		
				Relaxation (30 min × 2)		
				Outdoor exercises (45 min × 3)		
4	Six days in-patient stay with main focus on self-management (up to 16 patients within the same diagnostic group, 5 days with active treatment)	Rheumatologist, nurse, PT, OT, occupational assistant, SW, dietary supervisor, psychotherapist	None	Lectures concerning diseases and treatment, physical activity, nutrition, social security benefits, pain management, stress management, relaxation, communication, assessment of one’s own resources and limitations, values and choices, and personal goals	Consultations with team members as needed	Pool and gym exercising (as desired)
				Pool exercises (45 min × 1)		
				Gym exercises (60 min × 1)		
				Nordic walking (45 min × 1)		
5	Fifteen days in-patient rehabilitation (3 + 5 + 3 days with active treatment)	Rheumatologist, physician, PTs, OTs, SW, nurse	Twice pr week	Pool exercises (45 min × 1 or 2)	PT (45 – 60 min × 5)	Pool and gym exercising (as desired)
				Hand exercises (45 min × 5)	OT (as needed)	
					SW (as needed)	
6	Two weeks in- patient rehabilitation (5 + 5 days with active treatment)	Rheumatologists, nurse, PTs, OTs, occupational assistants, SWs, dietary supervisor, psychotherapist	Twice pr week	Pool exercises (30 min × 5)	PT (30 min × 5)	Pool and gym exercising (as desired)
					OT (30 min × 2)	
				Hand exercises (30 min × 5)	SW (as needed)	
					PT (as needed)	
				Activity pacing (60 min × 1)	Dietary supervisor (as needed)	
				Relaxation (15 min × 2)		
				Nordic walking (45 min × 2)		
				Gym exercises (45 min × 3)		

PT = physiotherapist, OT = occupational therapist, SW = social worker.

#### Intervention group

The intervention group will receive the new rehabilitation programme, which will be based on the existing rehabilitation programmes delivered at the participating centres, but with four additional elements:

1. A self-management booklet for use both during and after the rehabilitation stay;

2. A structured goal-setting process during the rehabilitation stay, including goals with which the patients will proceed in their home setting after discharge;

3. A follow-up programme consisting of four phone calls from a rehabilitation care provider at the rehabilitation centre directed at goal attainment and motivation for a continued effort;

4. Systematic use of motivational interviewing in the goal-setting process and in the follow-up phone calls.

Through the use of the following means, the aim of the new programme will be to solve ambivalence, increase self-efficacy, develop and maintain supportive self-talk, prevent the fading of achieved outcome and motivate for a continued effort by preventing relapse:

1. **The self-management booklet:** The booklet will be a personal belonging for the participant to keep and use both during and after the rehabilitation stay. It will be given to the participant at admission to the centre, and contain two main chapters. The first chapter will address topics of relevance for the rehabilitation stay such as “setting individual rehabilitation goals”, “motivation for change”, “positive self-talk”, “choosing what to pay attention to” and “worth remembering”.

The second chapter will contain topics of relevance for the first period after admission, starting with a summary of the first chapter. Thereafter, the headings will be as follows: “how to develop new goals”, “a retrospective look at motivation”, “one month after admission”, “three months after admission” and “windup”.

Throughout the book there will be open questions for the participant to answer, fields where he/she can make personal notes, small exercises in, e.g. mindfulness and relaxation, numeric rating scales where one can rate, e.g. one’s self-efficacy related to specific goals, quotes concerning rehabilitation goals, motivation and strategies taken from qualitative interviews with patients who have undergone rehabilitation [41] and illustrative pictures and poems.

The following templates will follow in an appendix, including a day- and week activity schedule, two 24-hour activity circles in which the participant can fill in current and desired activity pattern and an activity diary.

2. **The goal-setting process:** Patient-specific rehabilitation goals will be developed by the participant together with one or more members from the multidisciplinary team during an initial goal-setting conversation, and will be used as a basis for tailoring the rehabilitation interventions, while at the end of the rehabilitation stay the participants will be asked to evaluate their goals and choose the most important goals to proceed with in their home setting (see outline of questions in Table 2). Moreover, a follow-up support addressing strategies to achieve the individual rehabilitation goals and maintain self-management and adherence to health promoting behaviour will be discussed, and appointments will be made for the follow-up calls.

**Table 2:** An outline of the questions that will be asked in the initial goal-setting conversation a few days after admission, in the goal-setting conversation at discharge, and in the four follow-up telephone calls

**Question no.**	**Admission**
1	In this conversation we will try to agree on some goals for your rehabilitation stay. These should be based on what is important to you now and what you would like to change in your life, and they will be used to help you navigate during your stay and in your everyday life at home after discharge. So, could you tell me what thoughts you have concerning your long-term goals?
2	What would be important short-term goals to work on during your rehabilitation stay?
	**Discharge**
1	Let’s start with a brief look back. Is there anything you experienced or gained during your stay that has been particularly important to you?
2	What thoughts do you have about what should be your long-term goals now?
3	What will be your specific short-term goals? What will you do day by day and week by week to achieve your long-term goals?
4	And specifically, what will you do next week?
5	And tomorrow?
	**First telephone follow-up**
1	How are you?
2	Are there any specific challenges or issues you would like to discuss?
3	What are your most important goals now?
4	Could you please tell me a little about your plans for achieving those goals?
	**Second telephone follow-up**
	Same questions as in the first telephone follow-up call, with the following additional question:
5	Are there any other issues you’d like to discuss or new goals you’d like to pursue?
	**Third telephone follow-up**
	Same questions as in the second telephone follow-up call.
	**Fourth telephone follow-up**
	Same questions as in the second and third telephone follow-up, with the following additional question:
6	This is the last time I'll call you. Is there anything else you would like to add or discuss before we finish?

3. **Telephone follow-up calls:** After discharge, the patient will receive four telephone calls taking place one week after discharge, as well as one, three and five months after discharge, respectively (see outline of questions in Table 2).

4. **Motivational interviewing:** The therapeutic interventions offered in the rehabilitation intervention, the self-help booklet and the telephone follow-up calls will all be based on principles derived from cognitive behavioural therapy and MI [27,28].

Guides for how to perform the goal-setting process and follow-up telephone calls will be developed, including templates for recording the rehabilitation goals throughout the study period.

### Outcome measures

All outcome measures will be patient self-reported, and will be collected at admission and discharge, in addition to six and 12 months after discharge (see summary of measures to be collected in Table 3). The local study coordinator at each centre will administer the questionnaires to each participant after they have received oral and written information about the study and signed a written consent, and if needed, help and guidance on how to complete the questionnaires will be offered. At admission, the second set of questionnaires will be completed by the participant, and again, supervision will be offered.

**Table 3:** Summary of measures to be collected

	**Data collection instrument and scale**	**Time points**
**Primary outcome measure:**		
Goal attainment and health related quality of life	Patient generated Index (PGI) 0–100, 0 is good health	t1, t2, t3, t4,
**Secondary outcome measures:**		
Health related quality of life	Medical Outcome Study Short Form-36 (SF-36), 0–100, 100 is good health	t1, t2, t3, t4,
Pain	Numeric rating scale: 0–10, 0 is no pain	t1, t2, t3, t4,
Fatigue	Numeric rating scale: 0–10, 0 is no fatigue	t1, t2, t3, t4,
Global assessment of disease activity	Numeric rating scale: 0–10, 0 is no disease activity	t1, t2, t3, t4,
Motivation for change	Numeric rating scale: 0–10, 0 is no motivation	t1, t2, t3, t4,
Self efficacy pain	The Arthritis Self-Efficacy Scale (ASES) for pain, 1–5, 1 is low self-efficacy	t1, t2, t3, t4,
Self efficacy symptoms	The Arthritis Self-Efficacy Scale (ASES) for symptoms, 1–5, 1 is low self-efficacy	t1, t2, t3, t4,
**Cost-effectiveness:**		
Utility	SF6D utility index, 0.29–1.00, 1.00 is full health	t1, t2, t3, t4,
Sick leave and absence from work past six months	Number of days	t3, t4,
Health care utilisation past six months	Number of visits and/or hospital stays	t3, t4,
Use of medication	Name and dosage of medicine	t1, t2, t3, t4,
Medical or technical equipment past six months	Type and costs of equipment purchased	t3, t4,
Paid and unpaid help past six months	Costs and number of days and hours,	t3, t4,
Costs related to the rehabilitation stay	Costs pr day at each rehabilitation centre x number of days	t2
**Other measures**		
Age	Years	t1
Gender	Female/male	t1
Height	Centimetres	t1
Weight	Kilo	t1
Marital status	Living alone or not	t1
Employment status	Working full time/working part time/not working/student/working full time in the home/unemployed or seeking work/age retired/disability pension/sick leave	t1
Level of education	7-10 years of education, 10–12 years of education, more than 12 years of education	t1
Comorbidity	Presence of 16 diseases/health problems (yes/no)	t1
Level of exercising	3 times pr week, 1–2 times pr week, 1–2 times pr month, not on a regular basis, or I can not exercise or be social active due to functional limitations	t1
Physical and social activity	3 times pr week, 1–2 times pr week, 1–2 times pr month, not on a regular basis, or I can not exercise or be social active due to functional limitations	t1, t2, t3, t4
Physical activity	International Physical Activity Questionnaire-Short Form, IPAQ, mean minutes/week of, vigorous-intensity activity, moderate-intensity activity, walking, and sitting	t1, t2, t3, t4
Illness perception	Rheumatic Disease Illness Perception Questionnaire, 1 to 5, 1 is “not at all”	t1, t2, t3, t4

t1 = admission to rehabilitation, t2 = discharge from rehabilitation, t3 = six months after rehabilitation, t4 = twelve months after rehabilitation.

The data at the six- and 12-month follow up will be collected in telephone interviews by secretaries working at the National Resource Centre for Rehabilitation in Rheumatology. Two weeks before each interview, the questionnaires will be sent by post to the participant, together with a letter containing a suggested time for the interview and a request that the participant may read through- and complete the questionnaires on the last day before the interview. Contact information will be provided in the letter, and the participant will be encouraged to contact the secretary to make a new appointment if he/she is not available at the suggested time.

#### Primary outcome measure

The primary outcome in this study will be goal attainment and health-related quality of life measured by the Patient Generated Index (PGI) [42,43], which is an individualized instrument completed in three stages: In stage 1, the respondent will be asked to list up to five areas of life affected by his/her rheumatic condition, with an example list of areas provided that he/she can consult if necessary. A predefined sixth area will be listed as “all other areas of your life affected by your rheumatic disease”. In stage 2, the respondent will rate the areas given in stage 1 on numeric rating scales ranging from 0–6 where 0 = “as bad as could possibly be” and 6 = “as good as could possibly be”. In stage 3, the respondent will distribute 10 points to indicate the relative importance of each of the areas described in stage 1, with the most points allocated to the most important areas.

A summarized index score of PGI will be generated as follows: Each area ratings from stage 2 will be multiplied by the points given to the area in stage 3. Thereafter, the product of all areas will be summed up and divided by the number of areas (normally six including the predefined area). Lastly, this sum will be multiplied by 10 to yield a PGI index score between 0 and 100, with higher scores reflecting a better health-related quality of life.

At follow-up after six and twelve months, participants will be asked to score the same five areas that they listed in stage 1 at baseline.

In this study, the baseline PGI areas will also serve as a basis for developing individual rehabilitation goals for patients in the intervention group and for evaluating goal attainment.

The psychometric properties of the Norwegian version of the PGI has recently been tested with good results in patients with rheumatic diseases undergoing rehabilitation [39].

#### Secondary outcomes

A number of secondary measures will be included (see summary of measures to be collected in Table 3), and 11-point numeric rating scales (NRS) will be used to obtain self-reported pain, fatigue, global assessment of disease activity and motivation for change. A health-related quality of life will be measured by use of the Medical Outcome Study Short Form-36 (SF-36) [44], which is a widely used generic health measure. The SF-36 has eight subscales (physical functioning, role limitations due to physical problems, bodily pain, general health perceptions, vitality, social functioning, mental health, and role limitation due to emotional problems) that all contribute to two higher order health scales, the Physical Component Summary (PCS) and the Mental Component Summary (MCS) scores [44]. The Norwegian version of the SF-36 performs well in patients with rheumatoid arthritis [45], and has been used in previous studies examining the outcomes of rehabilitation in people with rheumatic diseases [4,46].

Self-efficacy is concerned with people’s judgments of their capabilities to execute given levels of performance and to exercise control over events [47], and will be measured using the sub-scales for pain and symptoms from the Arthritis Self-Efficacy Scale (ASES) [48]. The self-efficacy pain score is the mean of the statements for each sub-scale, and is expressed as a value between 1 and 5, with a score of 1 representing the lowest possible level of self-efficacy (Garrat A, Løchting I, Klokkerud M, Hagen KB: The Arthritis Self-Efficacy Scales (ASES) with five-point descriptive scales: results from a patient panel and survey of three rehabilitation centres, in preparation).

#### Cost-effectiveness

The direct and indirect costs in the study period will be self-reported at six and 12 months as the number of days of sick leave and absence from paid work over the past six months, the number of visits to a given list of health providers during the past six months and the number of hospital visits or stays over the past six months. The participants will also report on any medication taken for their rheumatic disease, medical or technical equipment purchased during the past six months and the number of days or hours they needed paid or unpaid help from others, including any costs related to paid help.

Additionally, data on health-care resource allocation and costs related to the rehabilitation stay will be collected at all centres. This will include recordings of the time used on the goal-setting conversations at admission and discharge, on the four follow-up calls, and on training of the involved personnel.

An important way of assessing the effects of treatment in health economic evaluations is the use of utility indexes, and in this study we will use the SF6D utility index as a utility measure in the cost-effectiveness analyses. SF6D is comprised of 11 items from the SF-36 [44] that are transformed into a continuous outcome scored on a 0.29–1.00 scale, with 1.00 indicating full health [49].

#### Other measurements

All participants will be asked to fill in a questionnaire at inclusion containing information on socio-demographics (age, gender, height, weight, marital status, employment and level of education), whereas comorbidity will be reported by asking the participant to check off the presence of 16 diseases/health problems (yes/no).

The level of exercising and social activity will be self-reported using two scales with the following response categories: “3 times per week”, “1-2 times per week”, “1-2 times per month”, “not on a regular basis” and “I cannot exercise or be socially active due to functional limitations”.

The level of physical activity will be self-reported using the International Physical Activity Questionnaire-Short Form, (IPAQ-SF), which is expressed as weekly energy expenditures determined by the expressed metabolic equivalent task minutes per week (METs min/wk) of different categories (sitting, walking, moderate- and vigorous-intensity physical activity and total physical activity score) and physical activity levels (low, moderate, and high) [50].

Illness perception will be captured by the Rheumatic Disease Illness Perception Questionnaire (RD-IPQ) [51], which is adapted for patients with rheumatic diseases from the original IPQ [52] and contains 11 questions about illness perceptions over the past two weeks, with five response alternatives ranging from “not at all” to “to a very large extent”. An overall scale of illness perceptions will be computed by adding the items representing illness consequences, illness emotions and illness identity. In a last open question, participants will be asked to write down any thoughts concerning the cause of their rheumatic disease.

### Text analyses

The content and stability of rehabilitation goals will be explored by text analyses, and the raw text materials for coding will be the rehabilitation goals agreed upon by the participants and health professionals during the semi-structured interviews shortly after admission, at discharge and in the follow-up telephone calls (see outline of questions to be asked in the interviews in Table 2).

The biostatistician will draw a random sample of approximately 25% of the participants from the total sample, stratified by centre and diagnosis.

A first analysis will be carried out separately by two independent researchers, who will read through the goal descriptions to identify and code statements concerning patients’ long- and short-term goals. Following this, the codes will be combined into broader categories, and finally into overarching themes [53]. Thereafter, the analyses will be compared and discussed until an agreement is reached, and if applicable, taxonomies for categorizing health and functioning such as the International Classification of Function, Disability and Health (ICF) will be used as a framework for the analyses [54]. Furthermore, goals agreed upon shortly before discharge or in the follow-up period will be compared to the initial goals to help explore whether goals are changing over time, and if there are trends or patterns that characterize such changes.

### Statistical analyses

A biostatistician (PM) blinded for group allocation will oversee the analyses of the data, with demographic and clinical characteristics, as well as other baseline data, presented to assess the baseline characteristics of the two groups, e.g. participants who have received the traditional rehabilitation programme (control group) versus participants who received the new programme (intervention group). These variables will also be compared for both those participants who withdraw from the study and those who remain. Parametric and non-parametric statistical analysis models will be used depending on the distribution of the variables.

The main comparative analyses between the two groups will be performed using an intention-to-treat (ITT) analysis, and descriptive statistics will be presented for each group as the mean change (standard deviation, 95% confidence intervals) in the outcomes from baseline to each time point. Because of the cluster nature of the randomisation, the differences in mean change will be compared between groups using Generalized Linear Repeated Measures Mixed models with random effects, adjusting for baseline values, a possible time trend and the clustering effects of centres [37,55]. The mixed model with random effect takes the clustering of the observations into account. In a linear mixed model with random effects the estimates will not always follow a F distribution. To assess the validity of the results, all p-values will therefore be estimated via the parametric bootstrap [56].

The main analysis for exploring factors associated with goal attainment and improved health-related quality of life during the first year following rehabilitation will be a multivariate manual backward stepwise linear regression, with the PGI sum-score at one year as the dependent variable. The number of variables included in the analysis will be kept below 10% of the included sample size, and will include age, gender, diagnosis, level of education, body mass index, comorbidity, pain, fatigue, disease activity, motivation for rehabilitation, level of exercises and physical activity, and illness perception.

All variables will be entered into the model and the best subsets of prognostic factors will be selected by excluding those independent variables with the smallest contribution to the model (i.e. those with the largest P-value). The model will be adjusted at every step for the baseline value of the outcome and the other independent variables.

The primary economic evaluation will take the form of a cost-effectiveness study of the cost per extra quality adjusted life years (QALYs), which will be calculated using the SF6D scores at six and 12 months, respectively. Differences in mean change from the baseline for the SF6D to each time point will be weighted by the time from the baseline using generalized linear regression modeling, adjusting for baseline levels of the SF6D to construct QALYs and then compared between groups.

### Ethical considerations

The study has been approved by the Norwegian Regional Committee for Medical Research Ethics (REK South-East, 2011/909) and by the Norwegian Social Science Data Services (2011/6602).

The research will be carried out in compliance with the Helsinki Declaration, personal confidentiality will be guaranteed and declarations of voluntary participation with detailed information on the study purposes and processes will be signed by each participant, thereby emphasizing the right to withdraw from the study at any time without any explanation.

The randomization procedure is deemed as being ethically acceptable because all participants will receive a rehabilitation stay comprised of either the rehabilitation programme currently provided at the participating centres or the new and potentially more effective programme. Hence, no participants will receive an intervention that is below the standard currently delivered at each centre.

## Discussion

Comprehensive rehabilitation, involving health professionals from various disciplines, is widely used as an adjunct to pharmacological and surgical treatment in people with rheumatic diseases. However, the evidence for the clinical- and cost-effectiveness of such interventions is limited [57].

This paper outlines the protocol for a study where the main aim will be to assess the effects of a structured goal planning and tailored follow-up programme in rehabilitation for patients with rheumatic diseases, using a multicentre, stepped-wedge randomized controlled design.

According to Brown et al. [37], two key conditions should be met in order to select the stepped-wedge design over a traditional parallel design: 1) There should be a prior belief that the intervention will do more good than harm, 2) There should be practical, logistical or financial constraints, which means that the intervention can only be implemented in stages. Both these conditions will be met in our study. Concerning the last condition, there would be a risk for contamination between two parallel groups localized at one institution in a traditional RCT because the patients in the groups might exchange information and experiences, thereby possibly diminishing the differences between the two intervention programmes. The stepped-wedge design protects against such contaminant effects.

Furthermore, all participants in the trial will receive rehabilitation, regardless of group allocation. This may reduce the chance that participants refuse participation for fear of ending up in a control group with an inferior treatment. The design may therefore increase the inclusion rate, and also to a large degree keep participants blinded for group allocation.

Based on current knowledge and evidence, we will develop a new rehabilitation programme that includes structured goal planning and tailored follow-up after discharge. The main objective of the study will be to evaluate goal attainment, health effects and the cost-effectiveness of this new programme compared to current traditional rehabilitation programmes. We will seek to include a minimum of 312 participants from six rehabilitation centres, which will make this one of the largest rehabilitation studies conducted within this patient population. The findings will therefore constitute an important contribution to more cost-effective- and evidence-based rehabilitation services for people with rheumatic diseases.

In a review of the efficacy of multidisciplinary team care programmes in rheumatoid arthritis, Vliet Vlieland et al. advocate the use of patient-oriented outcome measures and procedures to help enhance the role of the patient in the team care process and communication among health professionals [58]. The primary outcome in this study will be the Patient Generated Index (PGI), which is a patient-specific measure of a health-related quality of life that allows each patient to choose and rate the domains that he/she consider important.

Because the PGI captures the aspects of life that are of direct concern to the individual, the “noise” that is present in standardized instruments will be reduced, which in theory will have the potential to make it more responsive to capture the effects of rehabilitation [43]. In addition, the described domains may be used as a basis for discussing long- and short term rehabilitation goals, thus enhancing communication and an active role for the patient in the rehabilitation process.

A possible limitation in the PGI is that participants will select and score those areas of life that are experienced as problematic at baseline. The scores may therefore be prone to regression to the mean. As this probably will occur in both groups, it will, however, not affect the effect estimate, calculated as the differences between groups.

In this trial, we will also use the SF-36 as a standardised generic measure of health related quality of life. The combination of a patient-specific measure and a questionnaire with standardised items may ensure that different of aspects of health related quality of life are captured, and will also allow for comparison of populations and results across studies.

Regarding scientific benefits, the multicentre design will ensure that the new programme will be implemented in various settings, which enhances the generalizability of the results. Lastly, the study may increase the knowledge of how the structure, content and follow-up of rehabilitation stays can contribute in optimizing the effect of such stays. If proven effective, the programme may also be applied within other fields of rehabilitation, as well as in less complex interventions directed at lifestyle changes and self-management.

## Competing interests

The authors declare that they have no competing interests.

## Authors’ contributions

IK and KBH conceived the project idea and designed the study together with GB, IB, TD, AD, JH, BH, SGE, EF, MN, RWR, ALSS, BS and SHW. PM has performed the power calculations and determined the random order in which the different centres will switch from control to intervention period, and will perform the statistical analyses. IK, GB, IB, AD, BH, SGE, BS, and SHW administer the trial and coordinate assessments. TD, GB and IK have trained the health professionals involved in assessments and provision of the intervention. IB, AD, BH, SGE, BS and SHW recruit and screen the participants. IK has drafted this manuscript. All authors have provided feedback on drafts and have read and approved the final manuscript.

## Pre-publication history

The pre-publication history for this paper can be accessed here:

http://www.biomedcentral.com/1471-2474/15/153/prepub
